# Editorial: Thy1/CD90 Surface Glycoprotein: Sensor of Microenvironment?

**DOI:** 10.3389/fcell.2019.00162

**Published:** 2019-08-13

**Authors:** Emanuela Felley-Bosco, Lisette Leyton

**Affiliations:** ^1^Laboratory of Molecular Oncology, Department of Thoracic Surgery, University Hospital Zürich, Zurich, Switzerland; ^2^Programa de Biología Celular y Molecular, Cellular Communication Laboratory, Facultad de Medicina, Instituto de Ciencias Biomédicas, Universidad de Chile, Santiago, Chile; ^3^Advanced Center for Chronic Diseases, Center for Studies on Exercise, Metabolism and Cancer, Instituto de Ciencias Biomédicas, Facultad de Medicina, Universidad de Chile, Santiago, Chile

**Keywords:** membrane organizer, membrane micro domains, signaling platforms, cell adhesion molecule (CAM), Thy-1/CD90

Thy-1/CD90 is a small protein modified with lipids and carbohydrates that, as revised by Morris, represents up to 80% in molar terms of the total cell surface protein in thymocytes, from where its name derives. Indeed, a recent single cell transcriptome analysis of 20 mouse organs (The Tabula Muris Consortium et al., 2018) https://tabula-muris.ds.czbiohub.org/, indicates that immature T cells express overall the highest levels of Thy-1/CD90. Despite that, other cells express Thy-1/CD90, and several aspects concerning Thy-1/CD90 molecular interactions, signaling properties, and function in various cell types have been explored/reviewed in this Research Topic. This Frontiers in Cell and Developmental Biology issue includes novel data (Furlong et al; Ilic et al.; Picke et al.), as well as reviews and opinion papers (Jiang and Rinkevich; Hagood; Hu and Barker; Leyton et al.; Morris).

Thy-1/CD90 plays a role in lymphocytes differentiation. Furlong et al. investigate the impact of Thy-1/CD90 vs. T-cell-receptor (TcR) signaling on the production of the T helper (Th) cell subset-associated cytokines, and on the *in vitro* polarization of CD4+ T cells into Th1, Th2, and Th17 cells. The authors proposed the hypothesis that this antigen-independent activation may participate in host-to-host defense against extracellular pathogens. It would be of interest to explore whether this mechanism is involved in modulating immune cells in diseases escaping TcR-mediated T cell activation, such as low mutation tumor burden cancers.

Morris, a pioneer in exploring the function of Thy-1/CD90, calls Thy-1/CD90 “a prime exemplar of a membrane organizer” (Morris) because of its ability to influence and be influenced by its lipid environment. Leyton et al. places it as a “core of a membrane-associated platform (ThyMAP)” based on its capacity to interact with various proteins in the plane of the membrane, with proteins in membranes of opposite cells, and the associations of the multivalent complexes formed, with the extracellular matrix and the cytoskeleton. The regulation of Thy-1/CD90 by its surrounding lipids and proteins leads to fine tuning of the diverse physiological functions of Thy-1/CD90. An example of how the environment can influence Thy-1/CD90 localization is provided by Ilic et al. who, using biochemical assays, offer evidence that modification of a class of glycosphingolipids called gangliosides entails redistribution of Thy-1/CD90 in brain-derived neuronal membranes. In the neurological compartment, Thy-1/CD90 interacts with α_v_β_3_ integrin on reactive astrocytes to promote astrocyte adhesion (Leyton et al., 2001; Hermosilla et al., 2008; Lagos-Cabre et al., 2017). An interesting question would be whether this more adhesive phenotype may contribute to manifestation of diseases associated with ganglioside deficiency (Sandhoff et al., 2018).

An intriguing and yet unresolved aspect of Thy-1/CD90 is its expression in mesenchymal stem cells (MSC) and Hagood recalls that the expression of Thy-1/CD90 on MSC has been proposed to have several roles. MSC are for example considered precursor cells in bone formation (Chen et al., 2016) and Thy-1/CD90 acts as promoter of bone formation by positively regulating osteoblast differentiation and activation, while concomitantly inhibiting adipogenesis and obesity in mice (Picke et al., 2018). In this Research Topic, Picke et al. provide evidence that under conditions favoring obesity, lack of Thy-1/CD90 impairs bone formation while promoting bone resorption. This obese Thy-1/CD90^−/−^ condition is associated with (i) upregulation of tumor necrosis factor α and (ii) colony stimulating factor 1 (Csf1) expression, which are strong promoters of osteoclast differentiation, and (iii) a reduction in osteoprotegerin (Tnfrsf11b, decoy receptor of RANKL) expression, an inhibitor of osteoclast differentiation. In addition, MSC control the inflammatory response by contributing to the immunosuppressive environment (Estrela et al., 2017). As revised by Leyton's group in this Research Topic issue, this regulatory function of MSCs is correlated with high expression levels of Thy-1/CD90 (Leyton et al.).

Thy-1/CD90 is also expressed in fibroblasts, and Jiang and Rinkevich report that there are several populations of fibroblasts. These various cellular subsets are defined according to their anatomical site of origin and also on the basis of transient embryonic expression of genes, which program their ability to induce scar formation after wounding. In the skin, Thy-1/CD90 expression does not distinguish between fibroblasts with scarring ability and the other fibroblasts. In lung fibroblasts, the essential role in the regulation of mechano-transduction by integrin/Thy-1/CD90 interaction in response to increased or decreased matrix stiffness is reviewed by Hagood, while the same regulatory function in other cell types is reviewed by Hu and Barker. Additionally, in various inflammatory diseases, such as rheumatoid arthritis, systemic sclerosis, cancer, but not in cystic lung fibrosis, fibroblasts that overexpress Thy-1/CD90 have been related to disease progression (reviewed in Leyton et al.).

Contributing Authors point to some interesting facts and questions. For example, several years after providing the community with Thy-1/CD90 deficient mice (Nosten-Bertrand et al., 1996), Morris revised the influence of the genetic background on the phenotype. Sauzay et al. describe a possible role for the Unfolded Protein Response (UPR) in the regulation of Thy-1/CD90 expression. Other unanswered questions include, how the mode of anchorage of Thy-1/CD90 affects its signaling and function or the relevance of glycosylation in some of the seemingly discordant effects of Thy-1/CD90 in different contexts, or whether Thy-1/CD90 signaling has an effect on cell commitment.

In summary, Thy-1/CD90 functions as a microenvironment sensor that is important in pathological events, but the contributions of the molecule to disease development depend on the cellular context. Thus, using Thy-1/CD90 as a possible therapeutic target will require a better understanding of the positive and negative outcomes that inhibition of the molecule can lead to.

## Author Contributions

EF-B and LL were both guest editors of the Research Topic: Thy1/CD90 Surface Glycoprotein: Sensor of Microenvironment? and wrote this editorial.

### Conflict of Interest Statement

The authors declare that the research was conducted in the absence of any commercial or financial relationships that could be construed as a potential conflict of interest.
